# Supplementary figures and images for: Distracting stimuli evoke ventral tegmental area responses in rats during ongoing saccharin consumption
Source: Eur J Neurosci. 2021 Feb 2;53(6):1809–21. doi: 10.1111/ejn.15108 (PMC8603935; doi:10.1111/ejn.15108)

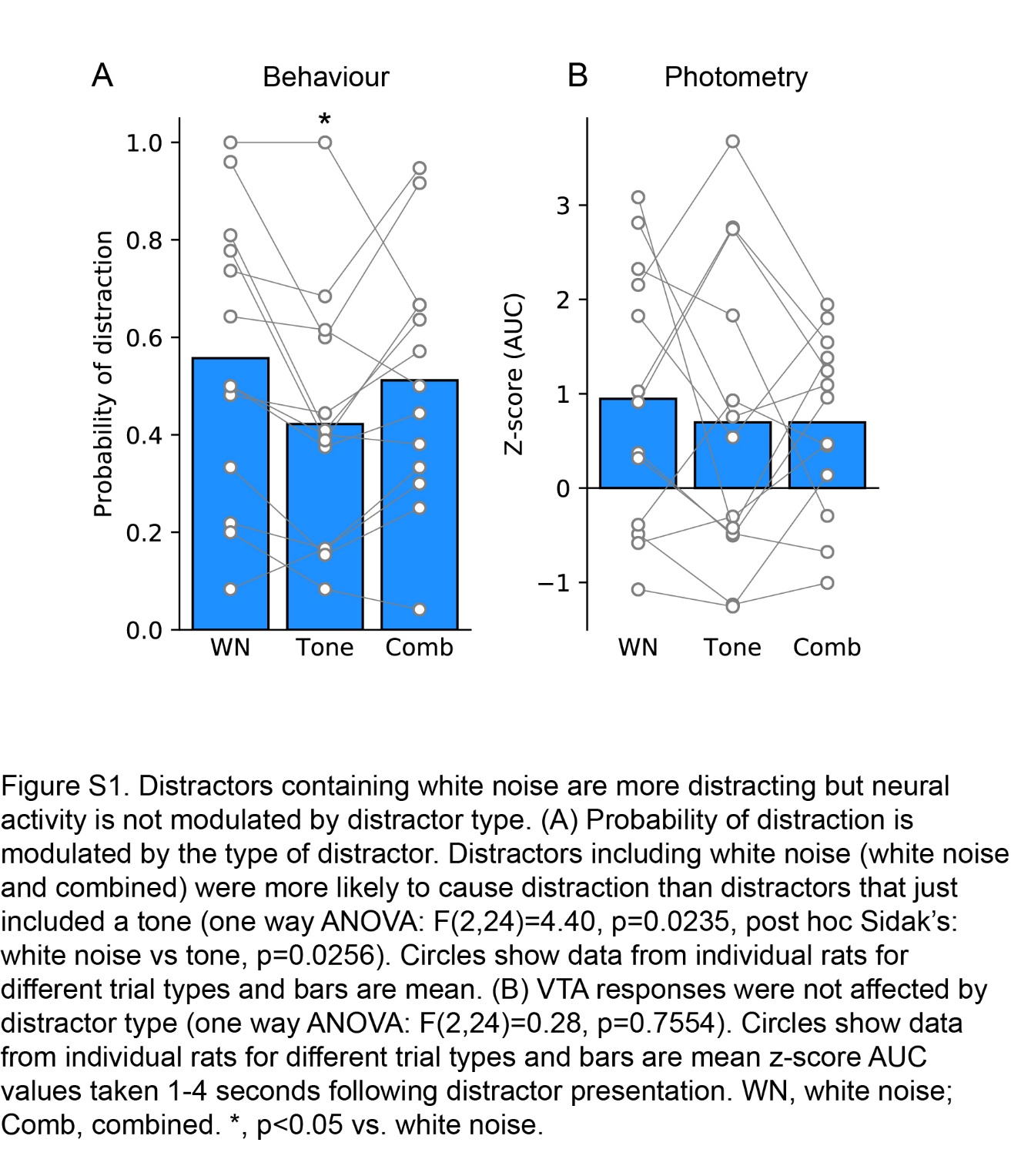


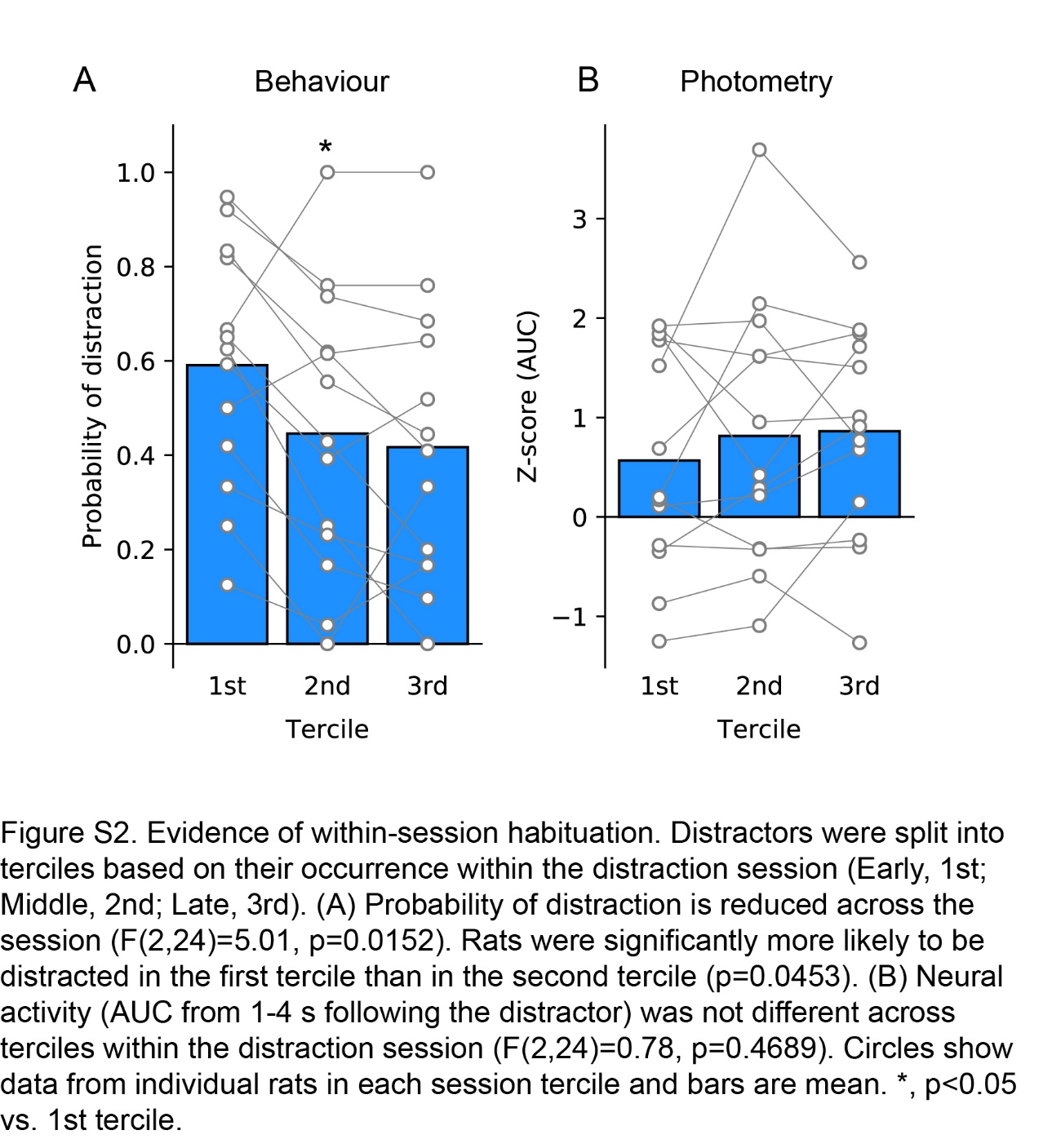


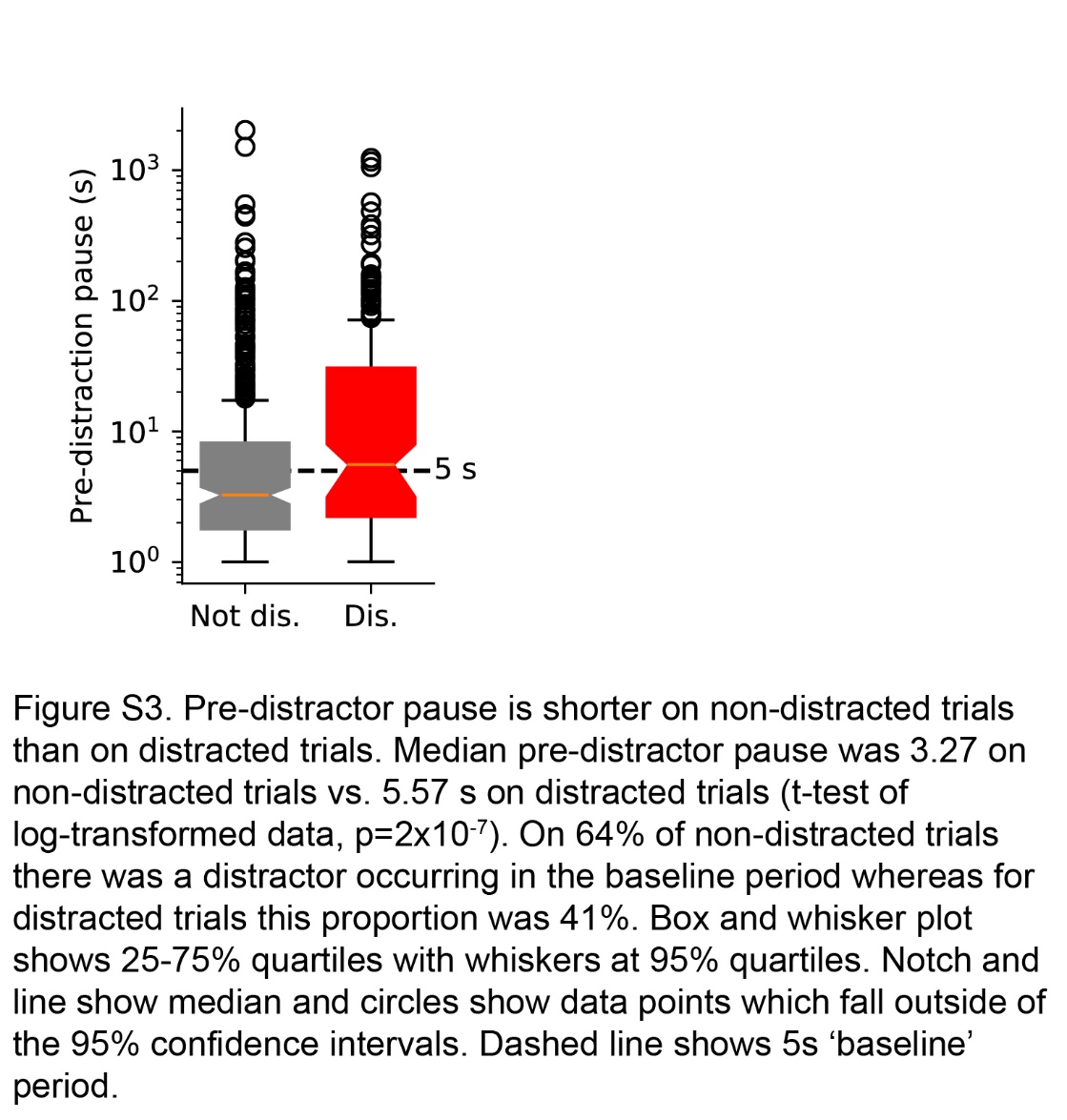


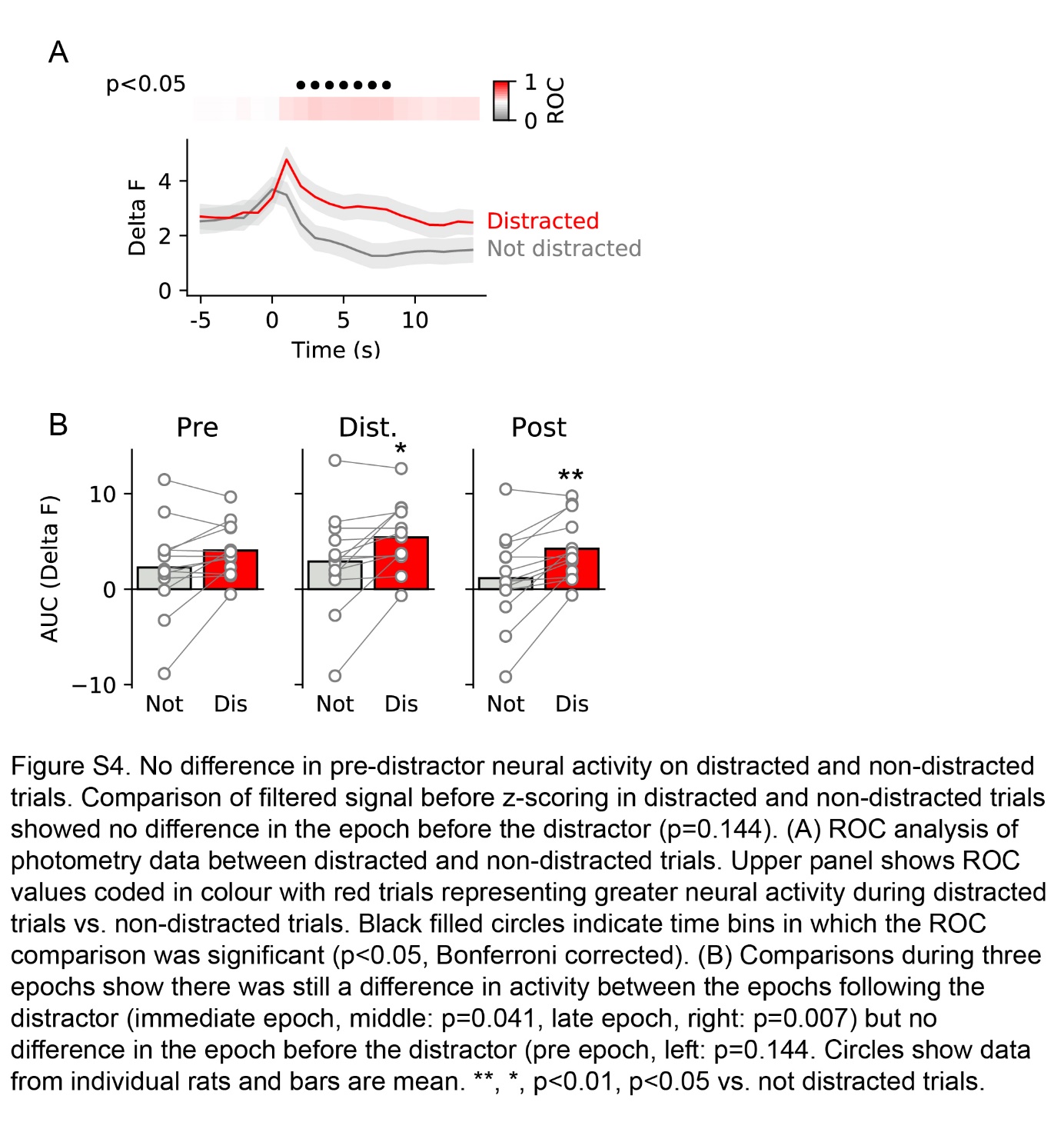


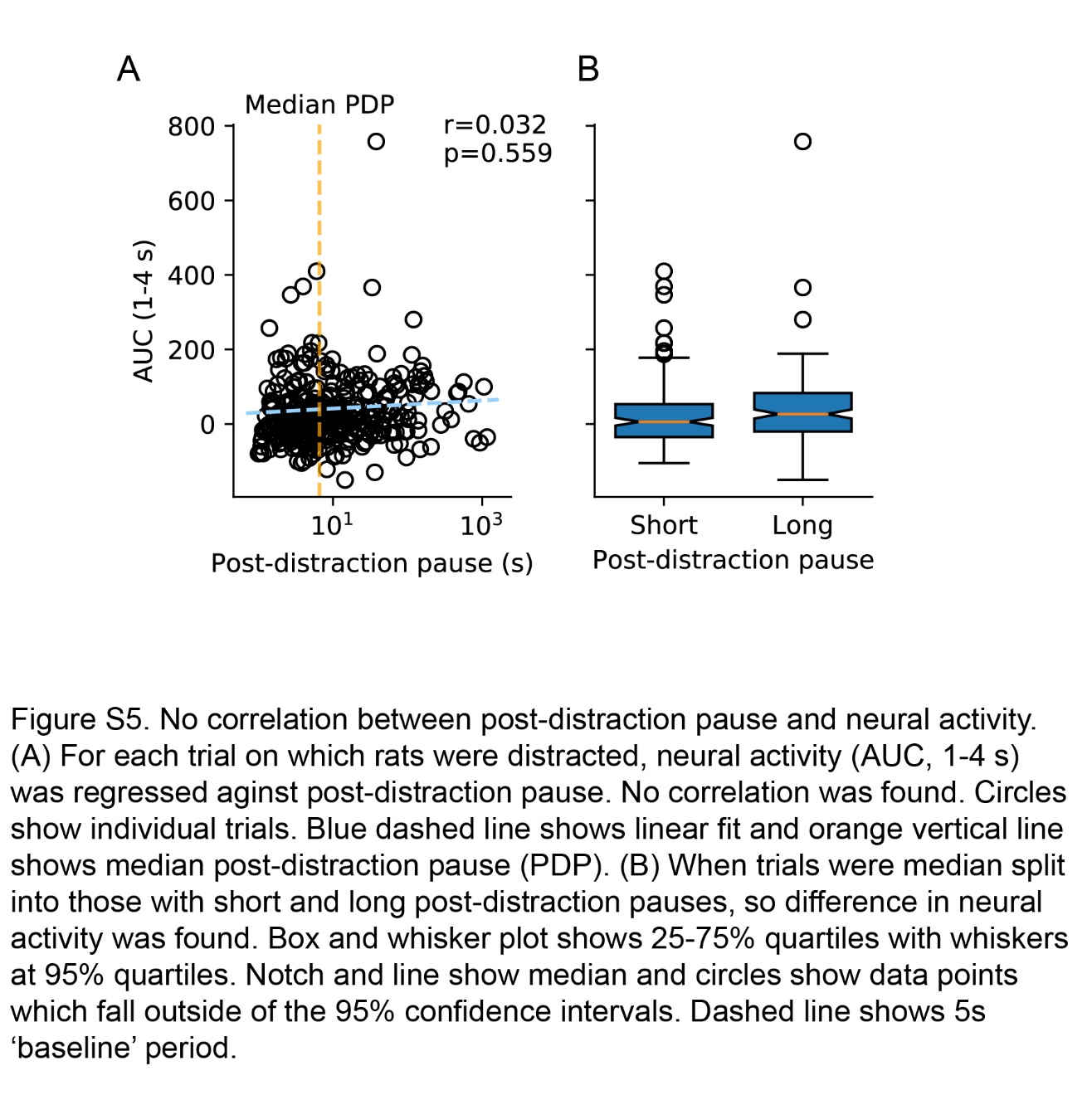


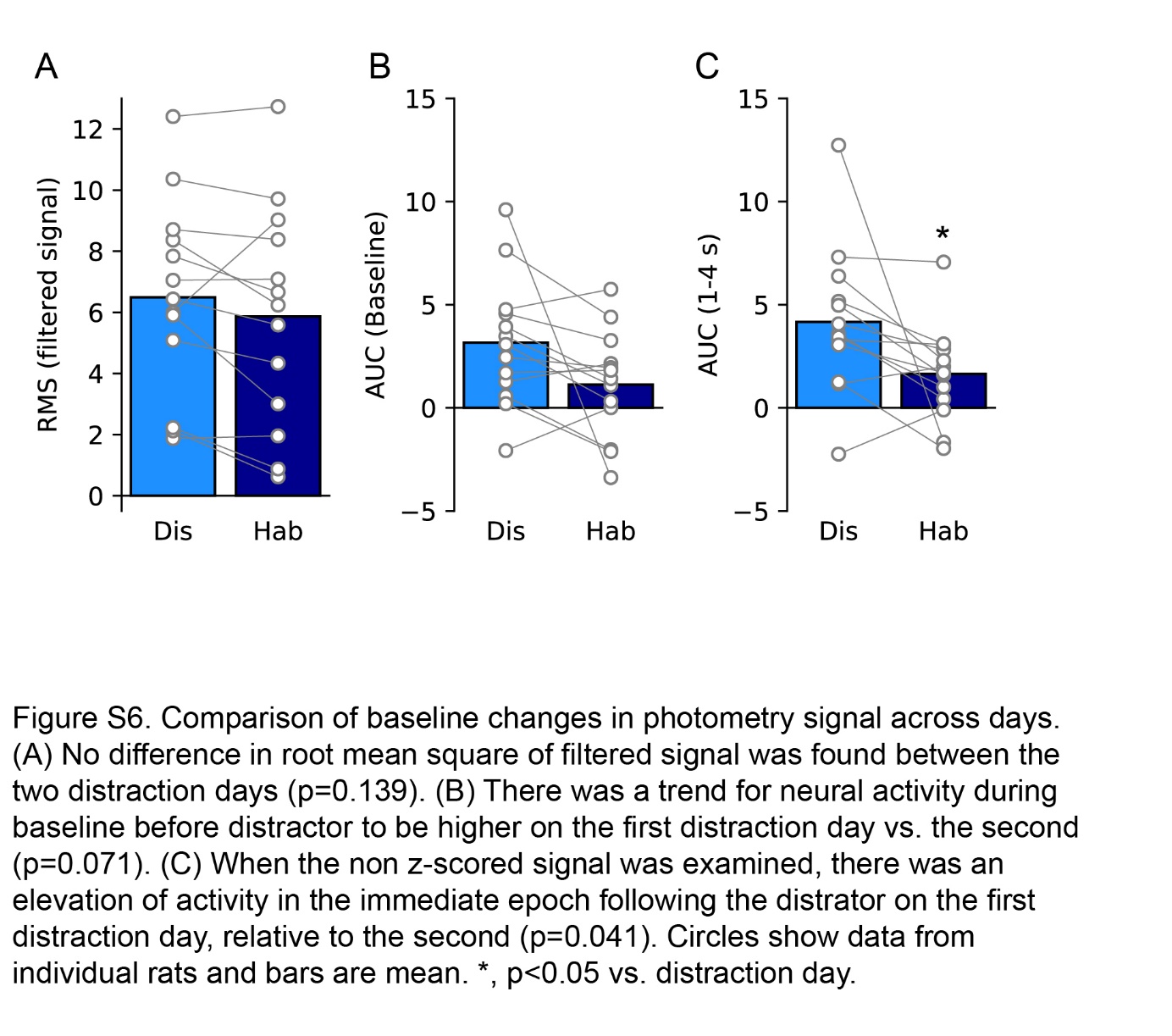

Supplement: Supplementary file 1 — Figure S1‐S6 [file EJN-53-1809-s001.docx]
